# Managing Prognosis in Precision Medicine: Utility, Imagination, and Communication

**DOI:** 10.3390/children10040664

**Published:** 2023-03-31

**Authors:** Rebecca Mueller, Brittany M. Lee, Katharine Press Callahan

**Affiliations:** 1 Department of Medical Ethics and Health Policy, University of Pennsylvania, Philadelphia, PA 19104, USA; 2 Masters Genetic Counseling Program, University of Pennsylvania, Philadelphia, PA 19104, USA; 3 Department of Pediatrics, University of Washington School of Medicine, Seattle, WA 98195, USA; 4 Seattle Children’s Research Institute, Seattle, WA 98101, USA; 5 Children’s Hospital of Philadelphia, Philadelphia, PA 19104, USA

**Keywords:** prognosis, communication, exome sequencing, psychosocial, prognostic utility, genomic, molecular profiling, ethics

## Abstract

Research on how physicians predict and communicate prognosis focuses primarily on end-of-life care. Unsurprisingly, as genomic technology gains traction as a prognostic tool, the focus has also been on terminality, with research focused on how genetic results may be used to terminate pregnancies or redirect care towards palliation for neonates. However, genomic results also have powerful impacts on how patients who live prepare for their futures. Genomic testing provides broad-reaching and early—albeit complex, uncertain, and shifting—prognostic information. In this essay, we argue that as genomic testing occurs earlier and increasingly in a screening context, researchers and clinicians must strive to understand and manage the prognostic implications of results. While our understanding of the psychosocial and communicational aspects of prognosis in symptomatic populations is incomplete, it has progressed further than our understanding in a screening context and therefore provides useful lessons and feasible opportunities for further research. By providing an interdisciplinary and inter-specialty perspective on the psychosocial and communicational aspects of prognosis in genetics, we discuss prognostication with respect to genetics from the neonatal period through adulthood, highlighting medical specialties and patient populations that are especially informative for considering the longitudinal management of prognostic information in genomic medicine.

## 1. Introduction

Precision medicine promises the right treatment at the right time [1]. But genomic testing also provides information that does not immediately direct treatment, instead providing information with respect to prognosis [2]. Indeed, research indicates that the perceived utility of genomic sequencing extends beyond clinical measures to behavioral, cognitive, emotional, and social parameters [3]. Yet healthcare researchers are only beginning to uncover the ways genetic information shapes expectations for patients’ futures at different points in life. As testing is increasingly deployed more widely, even in screening contexts to patients without known pathology, the impact of early testing must be studied to evaluate the effects of prognostic information.

Pediatric genomic medicine is premised on the idea that early diagnosis is optimal for patients and their parents. We argue that as genomic testing occurs earlier and increasingly in a screening context, researchers and clinicians must strive to understand and manage the prognostic implications of results. Extant research on how physicians predict and communicate prognosis focuses primarily on cancer and end-of-life care [4,5]. Unsurprisingly, as genomic technology emerges as a prognostic tool, the focus has also been on terminality. Researchers have studied how genetic results may be used to decide whether to terminate pregnancies [6] or redirect care toward palliation for neonates [7,8]. However, genomic results also have powerful impacts on how patients who live prepare for their futures. Genomic testing provides broad-reaching and early—albeit complex, uncertain, and shifting—prognostic information. More research is needed to understand how the prognostic implications of genetic results are managed over a lifetime.

Here, we provide an interdisciplinary and inter-specialty perspective on the psychosocial and communicational aspects of prognosis in genetics. While an understanding of the psychosocial and communicational aspects of prognosis in symptomatic populations is incomplete, it has progressed further than our understanding in a screening context and therefore provides useful lessons and feasible opportunities for further research. Looking across the lifespan, we discuss prognostication with respect to genetics from the neonatal period through adulthood. We highlight medical specialties and patient populations that are especially informative for considering the longitudinal management of prognostic information in genomic medicine and delineate future research in this domain.

## 2. From Prognostic Utility to Imagined Identities in the NICU

In the first weeks to months of life, babies emerge from anonymity. Parents begin to construct an identity and imagine a future for their children. A newborn participates in these processes through early forms of communication and interaction. Parents imagine additional interactions which add to the drama of the child’s identity formation [9]. Generally, identity is constructed on a backdrop of normality—children are predicted to have able bodies and able minds. The process by which parents develop identities for their newborns itself represents a type of prognostication, though it is not traditionally conceived as such.

When a newborn is critically ill, these processes of identity formation are radically disrupted. Parents struggle to feel like parents and to feel that they know their children [10]. They are often deprived of reciprocal interaction and even the basic activities of new parenthood: diapering, feeding, and holding. In place of these activities, information from clinicians becomes a charter for the future. Parents have a heightened awareness of prognostic questions, and diagnoses of all varieties take on prognostic power. Genetic diagnoses may be particularly potent in influencing parents’ sense of the future, as genetic essentialism is well documented in other contexts and increasingly recognized in neonatology [7,11]. Genetic diagnoses may be seen as more prescriptive or immutable than non-genetic ones, and even weak genetic explanations may be given undue weight [7,11].

This future-shaping effect of genetic diagnoses is frequently summarized with the catchphrase “ending a diagnostic odyssey” [12]. The term suggests the end of an arduous journey, presumably a traverse through the uncertain and lengthy search for a diagnosis. Our recent systematic review reported measures of utility in studies of genomic medicine for neonates [2]. Here too, the prognostic effects of genetic diagnoses are recorded as uniformly positive and are one of the most common benefits reported for genomic medicine. Yet, the reality of receiving prognostic genetic information early in life is more complicated. In the qualitative literature, genetic diagnoses made in the neonatal period take on different meanings for different families. For some families, a named diagnosis itself provides relief. In an interview study of parents undergoing rapid genome sequencing of their neonates, one parent explains that a diagnosis “gave us reassurance that as we made decisions for him, we really knew what was going on. And it also put a name to it, you know, versus saying our son has these symptoms and we don’t really know why. It gave us some closure as parents to know that there was a reason, that it’s been identified” [13] (p. 420).

Other parents report that early genetic diagnoses sometimes feel unnecessarily limiting and focused on pathology. In a narrative piece by this essay’s third author and Adams, a mother describes her experience receiving a Down syndrome diagnosis at birth: “[Henry] was immediately whisked away to be examined and monitored by a battery of specialists under the hospital’s Down syndrome protocol” (p. 2). In reflecting on her son’s birth, Adams was confident that specialists provided her son with the best possible care yet felt that their focus on discerning potential dysfunction resulted in unintended consequences as parents “come to know our children first, not as new members of our family but as potential sufferers of illnesses and delays” [14] (p. 2). This stands in contrast to the textured life Henry eventually develops, in which, despite his disability, he loves the minions and the lobsters at the grocery store and distracts his mom from homework by telling her she is beautiful. Another mother in the neonatal intensive care unit receives a diagnosis with a grimmer prognosis and highlights the problems of receiving prognostic information so early in infancy. She explains, “before all these tests, I think parents were able to cope better, get attached, smell the top of their kid’s head, feel like parents before being given a death warrant and the end of the story” and ultimately concludes that receiving this information later in her child’s life would have been more beneficial [15] (p. 1029).

Modern genomic medicine frequently generates uncertain prognostic information for neonates, which adds additional complexity [16]. Genetic results may be associated with a poorly defined phenotype, predictive of a broad range of outcomes, or be a variant of uncertain significance. Whole exome approaches to testing also reveal genetic variants associated with adult-onset disease risks or conditions. Given these longitudinal implications of genomic test results, the American Society for Human Genetics’ (ASHG) most recent statement on “Points to Consider: Ethical, Legal, and Psychosocial Implications of Genetic Testing in Children and Adolescents” recommends “that genetic testing in children should include a long-term communication plan for all results,” that considers who should be involved in such communication and when information should be shared “on the basis of age, maturity, and capacity to understand” [17] (p. 16). While the ASHG recommendations are apt and laudable, additional research is needed to determine the best practices for communicating genetic results and their evolving prognostic implications.

Whole-genome approaches to genetic testing raise new questions about communication because they afford a multitude of complex results, yet the challenge of comprehensible and developmentally appropriate communication has always been a part of the management of pediatric genetic conditions. In a reflective essay, Fanos compares the experience of the current cohort of “first families” adapting to novel diagnoses born of microarray analysis, whole-genome, and whole-exome sequencing to an earlier generation of families who first faced relatively new single-gene disorders in the middle of the 20^th^ century [18]. Similarly, we argue that a deeper understanding of the experiences of this earlier generation of patients with genetic diagnoses is critical to consider the benefits, harms, and communicational challenges of pediatric genomic sequencing.

## 3. From ‘Open Futures’ to Prognostic Imagination

Genetics research often focuses on the initial communication and reception of genetic test results [19,20,21], yet the stories told by affected individuals and their family members attune us to the lifelong challenge of prognostication. For example, disability activist and poet Laura Hershey learned the prognostic implications of her muscular dystrophy when she came across its definition in the dictionary. Sitting alone at school, she read: “A genetic disorder in which the body’s muscles weaken and eventually waste away.” At that moment, “All the futures I had imagined for myself were now replaced by this newly-revealed short future: ‘eventually, waste away’” [22] (p. 37). In contrast, genetic counselor Radhika Sawh always knew that her beta thalassemia major might shorten her life. Her older brother had died from it in childhood, and she grew up pioneering new treatments that had never been applied to young children. In her early twenties, when a boyfriend expressed serious intentions, Sawh recalls telling him, “I do not know anyone who is over 35 with this condition. You have to understand I don’t know how long I’m going to live. I don’t even know if I can have kids” [23]. Sawh’s story, along with Hershey’s, speaks to the complex challenges of communicating about and “living in prognosis” to use the words of anthropologist S. Lochlann Jain [24].

Jain theorized “living in prognosis” when grappling with “the firing squad of statistics” that came with their breast cancer diagnosis in their thirties [24]. While childhood-onset genetic conditions have altogether different temporalities than cancer in mid-life, these diagnoses also deploy a fusillade of data that may inflect time and future for both the affected individual and the whole family. The first author of this essay recently proposed prognostic imagination “to capture the complex ways that people envision their lives given diagnostic and prognostic information” [25]. This concept is key for genomic medicine because it conveys the impact of prognostic messages on what individuals envision, fear, dream, or plan for their lives in light of diagnostic and prognostic information [25]. At a technological moment when we often have access to more data than information, prognostic imagination calls for us to think critically about how we interpret and communicate the prognostic implications of genetic test results at diagnosis and as individuals grow into adulthood.

Despite the importance of prognostic communication, few studies investigate how children learn the prognostic implications of their diagnoses as they grow up and enter adulthood [26,27,28,29,30,31]. The few studies that do exist suggest that parents struggle to communicate with their children about prognosis and that both patients and parents want more information and assistance with respect to their futures [27,29,30]. Thus, before moving ahead with broadscale genetic testing in pediatrics—especially as screening—we need to take the challenge of prognostic communication across the lifespan seriously. This is especially important because pediatric genetic diagnoses often have variable and uncertain prognostic implications. Furthermore, prognoses are always evolving because our understanding of disease spectrums broadens as more people are tested [32] and because medical advances attenuate and alter disease processes. We need more research assessing how patients and families ascertain and internalize prognostic information to identify harmful consequences and provide the necessary support.

Take the example of cystic fibrosis (CF), a longstanding Mendelian disorder that was once considered lethal in childhood but now affects more adults than children in the United States [33]. Despite being relatively common, only a single paper discusses prognostic communication with affected individuals [27] outside of advanced care planning [34,35,36,37,38]. CF has been transformed by serial innovations from pancreatic enzymes, center-based care, and antibiotics [39] to targeted therapies [40]. With these therapeutic advancements, the life expectancy for CF has gradually increased from 5 years in 1954 [41] to 50 years in 2020 [40]. The recent introduction of cystic fibrosis transmembrane conductance regulator (CFTR) modulator therapies [40] both contributes to increasing life expectancy and highlights the fallacy of that metric. One woman with CF compared the impact of starting a CFTR modulator on her perception of the future to a cancer diagnosis in a “seemingly healthy person” [42]. Just as a terminal cancer diagnosis can replace “the possibility of a long-term future” with day-to-day living directed at reaching the next milestone, this young woman described already living day-to-day and then starting a drug that opened the “scary scenario” of “a future of endless possibilities” [42]. Thus, hopes associated with modulator therapies bring into relief the impact of early prognostic information on conceptions of time, future, and life plans. CF and other classical childhood onset-genetic conditions provide informative case studies for interrogating the psychosocial and communicational aspects of prognosis. By focusing on longstanding childhood-onset genetic conditions like CF, we can ask how affected individuals learn about prognosis initially and over time as the prognosis evolves. With that, we can develop an understanding of the impact of prognostic information, identify sources of misunderstanding, miscommunication, and misinterpretation, and establish communication guidelines and tailored psychosocial supports.

## 4. Lessons in Prognostic Communication from Pediatric Oncology

Because it is a relatively common biographical disruption that rapidly recasts one’s possible future, cancer has long been a focus in both scholarly work and lay perspectives on prognostication. In pediatric oncology, the heretofore open futures associated with childhood are abruptly threatened by a potentially terminal illness. Pediatric oncology has therefore been a prominent focus in normative and empirical work on communication and ethical issues in the care of children [43,44,45]. Extant research on prognostic communication in pediatric oncology may offer important guidance as we consider how to thoughtfully integrate genomics into pediatric medicine.

The clinical approach to prognostic communication in pediatric oncology has shifted over the prior half-century, as a diagnosis of childhood cancer has fortunately evolved from a universally terminal prognosis to a varied group of diagnoses with a median survival of 80% [46]. Nevertheless, the initial conversation between the pediatric oncologist and the parents of a child with cancer—the “Day One Talk”—continues to require careful attention to convey important information about diagnosis and treatment in an emotionally and informationally overwhelming setting [47]. When surveyed, most parents of children with cancer endorse valuing honest information about the prognosis of their child’s cancer—even when distressing—not only at the time of diagnosis but also throughout the course of their child’s treatment [48,49]. Honest, direct communication processes about prognosis can increase peace of mind, trust, and hope [46,50]. Likewise, in pediatric genomic medicine, many parents value receiving their child’s genomic testing results, even when uncertain in significance [51].

Yet despite these documented preferences in pediatric oncology, research indicates that pediatric oncologists may poorly estimate family’s informational needs, suggesting a need to explicitly assess each family’s individual needs [49,52,53]. A 2018 review of research on provider-parent/patient communication in pediatric oncology highlighted this generalizable lesson, noting that children and parents have persistent information needs throughout their illness journeys, but individuals can vary in the amount of information they desire [54]. Despite an increasing understanding of family needs and preferences about prognostic communication, there is plenty of space for improvement. Prognostic communication remains particularly challenging when patients have cancers with poor prognoses or especially when the prognosis is less certain. In these cases, studies have shown that pediatric oncologists temper their assessment of poor prognosis when speaking with families or avoid the discussion altogether, especially during periods of disease stability or when disease progression is unclear or equivocal [55,56,57]. Whether there are similar or different types of gaps in communication about the implications of genomic testing and results in children and neonates still needs to be explored, particularly in cases of clinical uncertainty or genetic results with uncertain significance.

Given the increasing data about parental preferences for communication in pediatric oncology, researchers have begun to propose methods to improve the longitudinal integration of prognostic communication into childhood cancer care. The “Day 100 Talk”, for example, is a structured conversation that takes place separate from the initial diagnostic disclosure and treatment discussions [58]. This intervention provides an explicit opportunity to focus on family concerns, with the goals of fostering open interdisciplinary communication and decreasing parental distress [58]. Interdisciplinary approaches may shift the responsibility of tracking and monitoring whether prognostic communication has occurred to the larger multidisciplinary clinical team rather than relying only on the patient’s primary oncologist [59]. Others have suggested communication strategies, such as “seed planting”, where prognostic information is offered gradually over time [60], or a “What If” framework to approach the discussion of uncertainties and explore concerns with families [61]. There is perhaps an opportunity to take similar approaches to both studying and enhancing prognostic communication with respect to genomic testing. As we increasingly supply families with prognostic information early in a child’s life, it is imperative to establish the key-time points at which to revisit genomic test results, elicit patient’s and parent’s questions and offer additional anticipatory guidance. Even in the context of classical childhood-onset genetic conditions, revisiting prognosis as a routine part of clinical care throughout a person’s life may be helpful as their ideas about it may change as medical advancements and their own medical trajectory unfold [25].

In addition to offering lessons on prognostic communication for genomic medicine more generally, pediatric oncology is also directly grappling with how to communicate information about genomic testing and its results alongside other diagnostic and prognostic results related to a child’s cancer diagnosis. In recent years, advances in technology have enabled the incorporation of more sophisticated molecular testing into clinical cancer care. Molecular sequencing of tumor cells can identify genetic alterations to employ molecularly-targeted therapies [62], which may be particularly useful for pediatric and young adult patients whose cancers do not respond to standard treatments [63]. Even when performed only on a child’s tumor cells, this testing has the potential to reveal a germline variant in the child—a genetic variant present both in the tumor cells and in the child’s healthy cells. Indeed, some centers routinely send paired tumor and “normal” samples to facilitate more easily distinguishing between variants present only in the tumor versus those in the germline. Extensive sequencing might therefore reveal secondary findings indicating a cancer predisposition in the patient, a predisposition for another adult-onset condition, or a variant of unknown significance (that may be reclassified over time). While the goal of the testing may be to assist in the diagnosis or management of the child’s cancer diagnosis, secondary or incidental genomic results may impact the child beyond their current diagnosis. The psychosocial impact of genomic testing on children with cancer and their parents is not entirely known. Advanced genomic testing of children with cancer is sufficiently new that recommendations for communicating this type of information have not yet been established. With time, the field of pediatric oncology will have to determine how to revisit genomic results as children grow up, enter survivorship, and establish adult care. In the meantime, pediatric oncology offers important models for considering the long-term process of prognostic communication in pediatric genomic medicine.

## 5. Towards Managing Prognosis across the Lifespan

Considering the potential impact of prognostic information across the lifespan will help us ask new and important questions about the psychosocial and communicational aspects of prognosis in genomic medicine and beyond. Because prognosis is an inherently longitudinal phenomenon, it is difficult to study amidst rapidly evolving genomic technologies. Accordingly, the genomics community will have to thoughtfully curate knowledge gleaned from different specialties and populations to achieve the call by ASHG for comprehensible and developmentally appropriate communication of genomic test results and to manage prognostic information across the lifespan. For example, by drawing on developmental work on parent-infant bonding, we can think critically about the potential impact of early applications of genomic testing on parents’ perceptions of their children’s future and design studies to assess them. Similarly, Fanos’ description of an earlier group of “first families” of genetics attunes us to the generations of patients and parents who have already lived through decades of prognostic uncertainty [18], whose previously documented experiences can be mined for important insights. Lastly, pediatric oncology can provide us with models for how to study prognostic communication to work toward the development of communication tools and timelines. Integrating and extending these types of research can equip us to do our best for our patients and their families as modern technology enables more and earlier genetic diagnoses.

Moving forward, it will be critical to design studies aimed at assessing the longitudinal impact of prognostic information across the lifespan. Studies assessing how parents and patients report ascertaining prognostic messages beyond the initial diagnosis will establish the timing and sources of prognostic messages that influence families’ understandings and perceptions of prognosis. Interviewing patients who have received prognostic genetic information or clinicians who have delivered this information at different time points would add perspective. Longitudinal research is also necessary to assess patient and parent experiences at different time points in relation to either the timing of diagnosis or the proband’s age or life stage. Importantly, these studies must extend beyond pediatric populations to enroll affected adults of different ages and bereaved family members of affected individuals who passed away from their diagnoses. Collectively, such work can provide the field of genomic medicine with a nuanced view of the longitudinal impact of prognoses that inevitably evolve over a lifetime.

Whether counseling new parents of an infant in the NICU, caring for an adult navigating prognostic uncertainty alongside life decisions, or addressing a child and parents facing an adult-onset genomic finding coincident with a cancer diagnosis, we must communicate about genetic diagnoses with an eye towards managing prognosis across the lifespan. Attending to the longitudinal experiences of affected adults and parents of affected children can help to move the field beyond prognostic utility to establish how prognoses may shape imagined futures and how prognostic imagination inflects hopes, fears, decisions, and plans [25]. In doing so, clinicians and researchers can learn how best to communicate with families as individuals age and prognoses evolve. Furthermore, such work can provide insights that aid in assessing the potential benefits, harms, and communicational challenges associated with the application of sequencing as a screening tool in apparently healthy fetuses and newborns. Understanding the impact of early prognostic information takes a lifetime and therefore requires novel integrative approaches to keep pace with the burgeoning applications of genomics in pediatric medicine. Rapid sequencing has long-lasting consequences.

## Data Availability

No new data were created to write this essay.
